# Supplementary material for: The causal relationship between the human gut microbiota and pyogenic arthritis: a Mendelian randomization study
Source: Front Cell Infect Microbiol. 2024 Nov 26;14:1452480. doi: 10.3389/fcimb.2024.1452480 (PMC11629706; doi:10.3389/fcimb.2024.1452480)
Supplement: Supplementary file 1 [file DataSheet1.zip › mendelian test/ebi-a-GCST90027484.csv_leave_one_out.pdf]

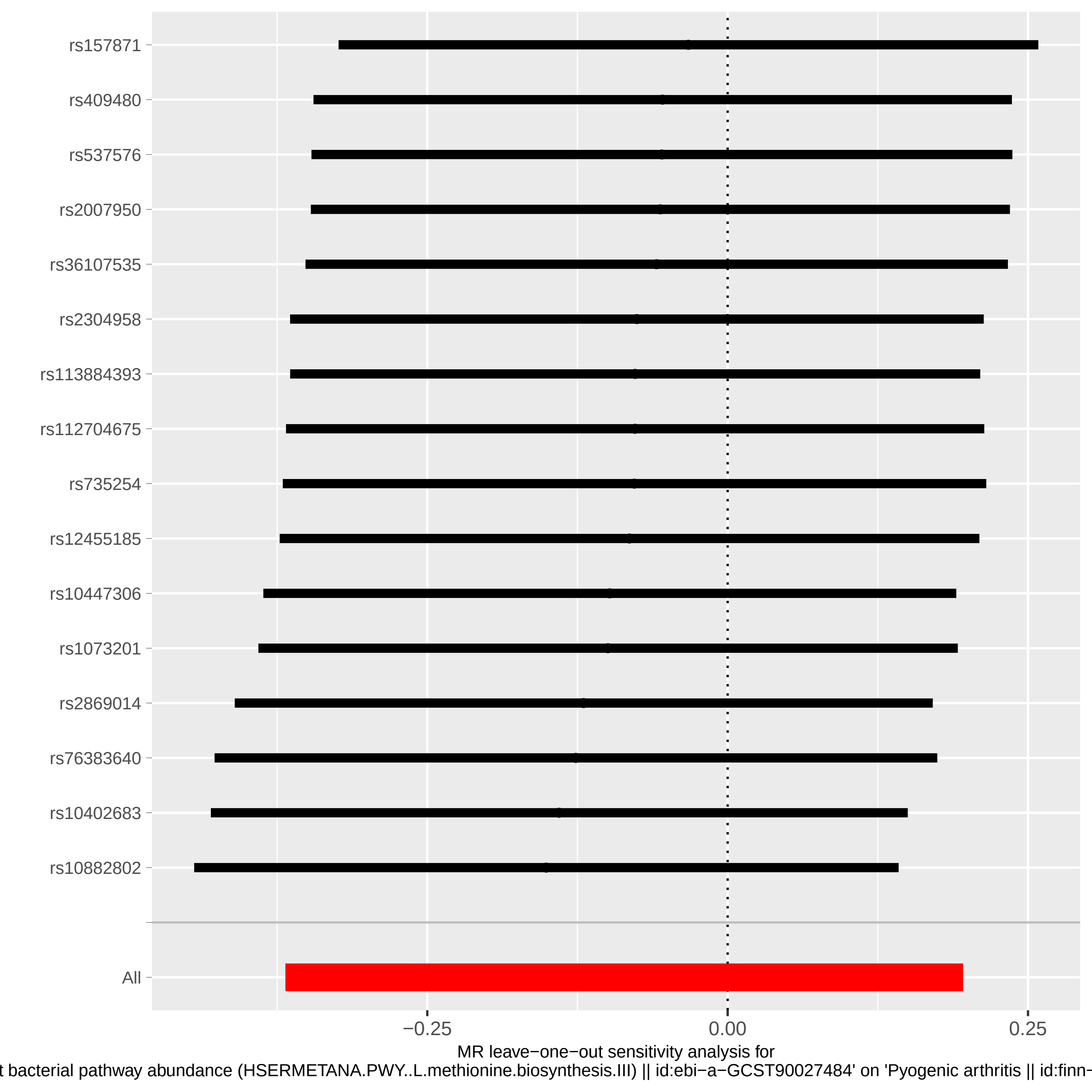
rs157871
rs409480
rs537576
rs2007950
rs36107535
rs2304958
rs113884393
rs112704675
rs735254
rs12455185
rs10447306
rs1073201
rs2869014
rs76383640
rs10402683
rs10882802
All
−0.25
0.00
0.25
MR leave−one−out sensitivity analysis for
t bacterial pathway abundance (HSERMETANA.PWY..L.methionine.biosynthesis.III) || id:ebi−a−GCST90027484' on 'Pyogenic arthritis || id:finn−
